# Supplementary material for: Infliximab Efficacy May Be Linked to Full TNF-α Blockade in Peripheral Compartment—A Double Central-Peripheral Target-Mediated Drug Disposition (TMDD) Model
Source: Pharmaceutics. 2021 Nov 1;13(11):1821. doi: 10.3390/pharmaceutics13111821 (PMC8623740; doi:10.3390/pharmaceutics13111821)
Supplement: Supplementary file 1 [file pharmaceutics-13-01821-s001.zip › pharmaceutics-1400655-supplementary.pdf]

pharmaceutics

Supplemental Materials: Infliximab Efficacy May Be Linked to Full TNF-α Blockade in Peripheral Compartment—A Double Central-Peripheral Target-Mediated Drug Disposition (TMDD) Model

David Ternant, Olivier Le Tilly, Laurence Picon, Driffa Moussata, Christophe Passot, Theodora Bejan-Angoulvant, Céline Desvignes, Denis Mulleman, Philippe Goupille and Gilles Paintaud

1. Sensitivity analysis for unbound target elimination rate constant (k_{out})

Raw infliximab concentrations for inflammatory bowel disease patients and ankylosing spondylitis patients can be found in figure S1.

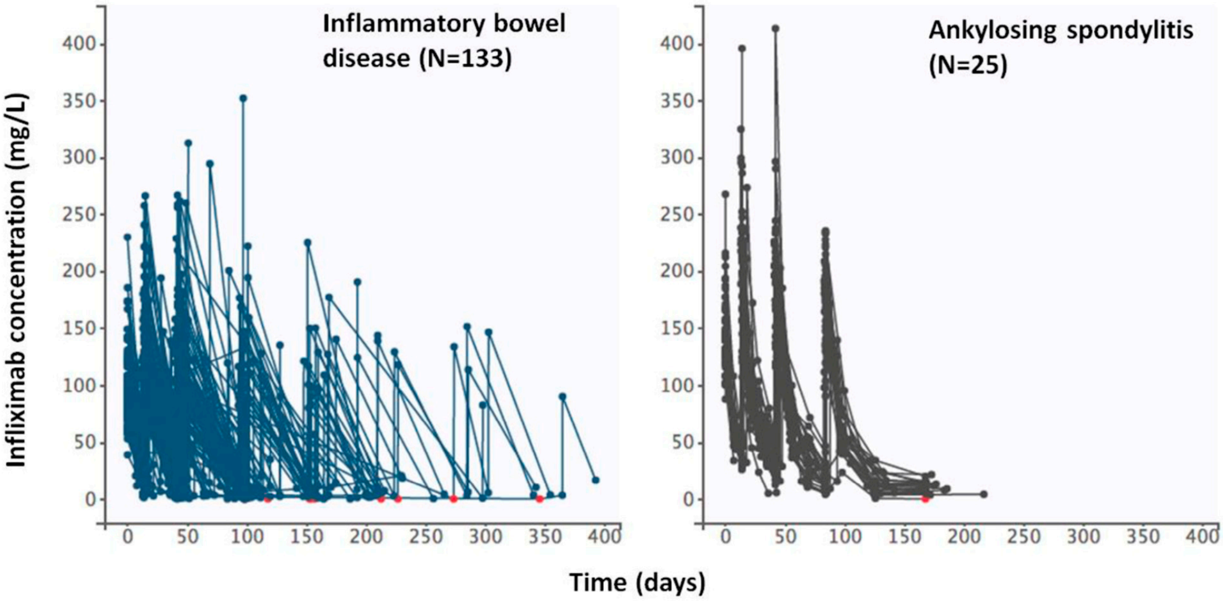

Figure S1. Spaghetti plots representing observed infliximab concentrations in time for inflammatory bowel disease patients (left) and ankylosing spondylitis patients (right). Red points are concentrations below the lower limit of quantitation (0.103 mg/L).

2. Sensitivity analysis for unbound target elimination rate constant (k_{out})

Based on these possible values, the double QSS model was run while fixing k_{out} at the following values: 5, 10, 20, 40, 100, 150 or 200 day^{-1}. The value leading to best model performances was retained. This was done on the central TMDD model and verified on the double TMDD model while fixing the same k_{out} value for both central and peripheral compartments. Indeed, AIC decreased between 5 and 200 day^{-1}, but values from 40 to 200 day^{-1} led to difficulties in providing acceptable parameter estimation accuracy (table S1), with r.s.e values above 60% for k_{int} and K_{SS} estimates.

Table S1. Selection of fixed TNF-α elimination rate constant values.

Model	k_{out} value (day^{-1})	5	10	20	40	60	80	100	200
1QSS base	−2LL	10,827.89	10,818.4	10,818.28	10,817.97	10,816.65	10,816.15	18,815.47	10,813.99
	AIC	10,853.89	10,844.4	10,844.28	10,843.97	10,840.65	10,842.15	18,839.47	10,839.99
	Accuracy	Good	Good	Good	Average	Average	Average	Average	Poor
2QSS base	−2LL	10,801.33	10,796.2	10,793.76	10,792.54	10,792.01	10,791.41	10,789.97	10,788.14
	AIC	10,835.33	10,830.2	10,827.76	10,826.54	10,826.01	10,825.41	10,823.97	10,822.14
	Accuracy	Good	Good	Good	Average	Average	Poor	Poor	Poor

Accuracy refers to quality of parameter estimation measured by relative standard error (r.s.e) and variance-covariance matrix of estimates. **Legends:** QSS: Quasi-steady-state, *−2LL: −2 ln-likelihood; AIC: Akaike's information criterion,* k_{out}: TNF-α elimination rate constant.

3. Modified TMDD model

The present study showed that the double central-peripheral target-mediated drug disposition (TMDD) model with quasi-steady-state (QSS) approximation led to a better description of infliximab concentration-time data than basic two-compartement model or two-compartment model with TMDD in central or peripheral compartments. However, the double TMDD model assumed no transfer of unbound TNF-α between central and peripheral compartments. Therefore, we attempted several modeling approach to account for TNF-α transfers between compartments. In this section, we present our best strategy (figure S2).

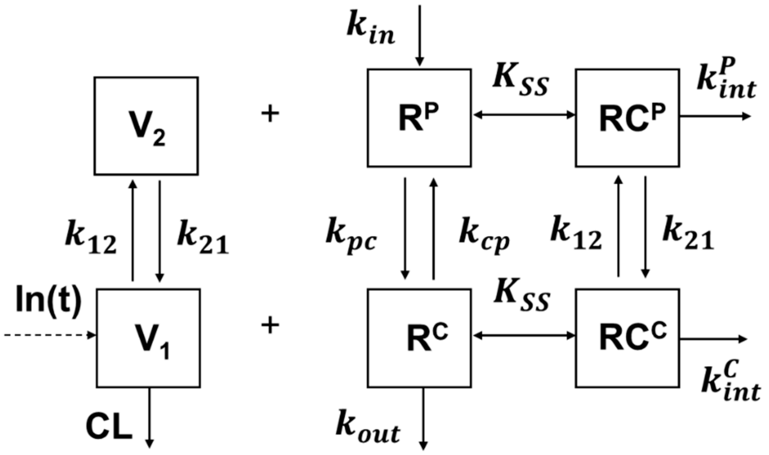

Figure S2. Target-mediated drug disposition (TMDD) with quasi-equilibrium (QSS) approximation. This model assumes TNF-α input (k_{in}) and output (k_{out}) in central and peripheral compartments, respectively, and bidirectional exchanges of unbound TNF-α (k_{pc} and k_{cp}). Transfer of infliximab- TNF-α complexes is assumed to be driven by infliximab transfer kinetics (k_{12} and k_{21}). While elimination rate constant of complexes are supposed different in central (k_{int}^{C}) and peripheral (k_{int}^{P}) compartments, steady-state (K_{SS}) dissociation constant is supposed to be of the same value in both compartments.

The modified TMDD model is based on the following assumptions:

- TNF-α zero-order input (k_{in}) and first-order output (k_{out}) in central and peripheral compartments, respectively;
- First-order intercompartment bidirectional exchanges of unbound TNF-α (kpc and kcp);
- Intercompartment transfer of infliximab-TNF-α complexes are bidirectional and are fixed by infliximab intercompartment transfer rate constants (k12 and k21);
- Different elimination rate constants of infliximab-TNF-α between central and peripheral compartments;
- Same value of quasi-steady-state dissociation constant (KSS) in both compartments.

Based on these assumptions, the modified TMDD model was:

$$\frac{dC_T}{dt} = In(t) - \frac{CL}{V_1}.C - k_{12}.C_T + k_{21}.C_{PT} - k_{int}^C.(C_T - C)$$

$$\frac{dR_T^C}{dt} = k_{in}^C - k_{cp}.(R_T^C - C_T + C) + k_{pc}.(R_T^P - C_{PT} + C_P)$$

$$-k_{12}.(C_T - C) + k_{21}.(C_{PT} - C_P) - k_{int}^C.(C_T - C)$$

$$C = \frac{1}{2}\left[(C_T - R_T^C - K_{SS}^C) + \sqrt{(C_T - R_T^C - K_{SS}^C)^2 - 4.K_{SS}^C.C_T}\right]$$

$$\frac{dC_{PT}}{dt} = k_{12}.C_T - k_{21}.C_{PT} - k_{int}^P.(C_{PT} - C_P)$$

$$\frac{dR_T^P}{dt} = k_{cp}.(R_T^C - C_T + C) - k_{pc}.(R_T^P - C_{PT} + C_P)$$

$$-k_{12}.(C_T - C) - k_{21}.(C_{PT} - C_P) - k_{out}.(R_T^P - C_{PT} + C_P) - k_{int}^P.(C_{PT} - C_P)$$

$$C_P = \frac{1}{2}\left[(C_{PT} - R_T^P - K_{SS}^P) + \sqrt{(C_{PT} - R_T^P - K_{SS}^P)^2 - 4.K_{SS}^P.C_{PT}}\right]$$

where In(t) is infliximab input function, C and C_T are unbound and total infliximab concentrations in central compartment, respectively, C_P and C_{PT} are unbound and total infliximab concentrations in peripheral compartment, respectively, V_1 and V_2 are central and peripheral volumes of distribution, respectively, CL and Q are systemic and intercompartmental clearances, respectively, R_T^C and R_T^P are latent total TNF-α levels interacting with infliximab in central and peripheral compartments, respectively. To initiate the system, we estimated global target antigen amount as follows: $R_0 = R_0^C + R_0^P$, with $R_0^C = k_{in}/k_{out}$ and $R_0^P = \frac{k_{in}}{k_{cp}} + k_{pc}.\frac{k_{in}}{k_{out}}$.

The modified TMDD model led to satisfactory description of infliximab concentration-time data: parameter estimates were of acceptable accuracy (table S2) and no structural parameter needed to be fixed. Visual predictive checks (VPC) and normalized prediction distribution errors (NPDE) showed no obvious bias or model misspecification (figure S3). Even if this model did not necessitate fixing one structural parameter value, it presented lower performances than our final double TMDD model in terms of AIC (10,813.05 vs. 10,792.05 for modified TMDD vs. final double TMDD models, respectively). In addition, k_{pc} and k_{int}^P were not estimated with good accuracy (table S2). We believe that a sound mechanistic description of TNF-α turnover would be valuable and should be applied on datasets with dense concentration-time data.

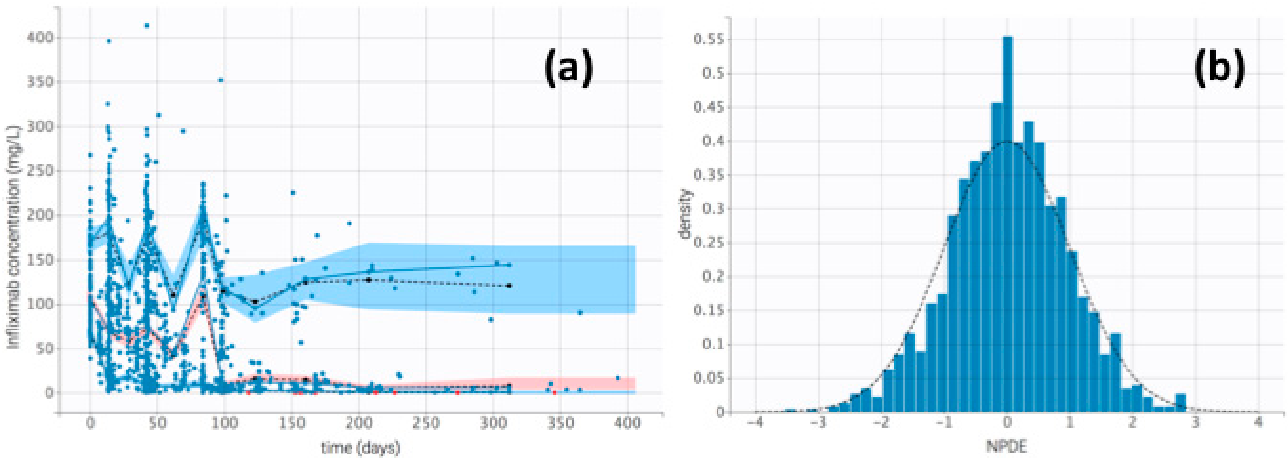

Figure S3. Diagnostic plots of the modified TMDD model. **(a)** normalized prediction distribution error (NPDE) distribution vs. Gaussian probability density function; dashed line is theoretical Gaussian distribution; **(b)** visual predictive check; observed concentration (black circles), theoretical (dashed lines) and empirical (continuous lines) percentiles (from

bottom to top: 10%, 50% and 100% percentiles) and prediction interval (from bottom to top: 10%, 50% and 90% prediction intervals.

Table S2. Parameter estimates for final modified TMDD model.

Parameter	Unit	Estimate	RSE%
V_1	L	2.7	3.3
CL	L.day^{-1}	0.14	8.6
k_{12}	L	0.19	19
k_{21}	L.day^{-1}	0.29	20
R_0	nM	26.8	23
k_{cp}	day^{-1}	1.1	51
k_{pc}	day^{-1}	0.072	43
K_{SS}	nM	9.0	20
k_{out}	day^{-1}	4.3	14
k^{C}_{int}	day^{-1}	0.057	12.3
k^{P}_{int}	day^{-1}	0.0028	54.9
BW_V_1	—	0.28	38
SX_V_1	—	0.11	42
SX_CL	—	0.39	25
ω_{V1}	—	0.25	6.9
ω_{CL}	—	0.29	15
ω_{k21}	—	0.68	17
ω_{R0}	—	0.71	23
ω_{kcp}	—	1.1	28
σ_{add}	mg/L	1.8	7.7
σ_{prop}	—	0.20	2.8
−2LL	—	10,771.05	—
AIC	—	10,813.05	—

Legends. TMDD: target-mediated drug disposition, V_1: central volume of distribution, CL: systemic clearance, k_{12}, k_{21} central-to-peripheral and peripheral-to-central infliximab transfer rate constants; K_{SS}: steady-state dissociation constants relative to central and peripheral compartments, R_0: baseline TNF-α amount, k_{int}: infliximab-TNF-α complex elimination rate constant, "C" and "P" stand for central and peripheral compartments, respectively; k_{out}: TNF-α elimination rate constant, *t*BW: body weight, SX: sex, UC: ulcerative colitis, *−2LL: −2 ln-likelihood; AIC: Akaike's information criterion.*
